# Activation of TRPV4 by lactate as a critical mediator of renal fibrosis in spontaneously hypertensive rats after moderate- and high-intensity exercise

**DOI:** 10.3389/fphys.2022.927078

**Published:** 2022-09-08

**Authors:** Binyi Zhao, Yanping Xu, Yunlin Chen, Ying Cai, Zhiyan Gong, Dan Li, Hongyu Kuang, Xiaozhu Liu, Hao Zhou, Guochun Liu, Yuehui Yin

**Affiliations:** ^1^ Department of Cardiology, The Second Affiliated Hospital of Chongqing Medical University, Chongqing, China; ^2^ Department of Infectious Diseases, Institute for Viral Hepatitis, The Second Affiliated Hospital, Chongqing Medical University, Chongqing, China; ^3^ Department of Ultrasonography, The First Affiliated Hospital, Chongqing Medical University, Chongqing, China; ^4^ The College of Exercise Medicine, Chongqing Medical University, Chongqing, China

**Keywords:** SHR (spontaneous hypertensive rat), exercise, TRPV4 (transient receptor potential vanilloid 4), lactate, renal, fibrosis

## Abstract

Moderate-intensity exercise training has been regarded a healthy way to alleviate kidney fibrosis by the transforming growth factor-beta (TGFβ) signaling pathway. However, the impact of different intensity exercise training on renal function is unknown, and the underlying mechanism is also unclear. The purpose of this study is to explore the effect of lactic acid in different intensity exercise training on renal fibrosis in spontaneous hypertension. Masson’s trichrome staining, immunohistochemistry, lactic acid kit, and Western blotting were applied on the excised renal tissue from six male Wistar–Kyoto rats (WKY) and 18 male spontaneously hypertensive rats (SHR), which were randomly divided into a sedentary hypertensive group (SHR), moderate-intensity exercise hypertensive group (SHR-M), and high-intensity exercise hypertensive group (SHR-H). The results revealed that renal and blood lactic acid, as well as the key fibrotic protein levels of transient receptor potential vanilloid 4 (TRPV4), TGFβ-1, phospho-Smad2/3 (p-Smad2/3), and connective tissue growth factor (CTGF), were significantly decreased in the SHR-M group when compared with the SHR and SHR-H groups. In further *in vitro* experiments, we selected normal rat kidney interstitial fibroblast (NRK-49F) cells. By immunofluorescence and Western blotting techniques, we found that TRPV4 antagonists (RN-1734) markedly inhibited lactate-induced fibrosis. In conclusion, compared with previous studies, high-intensity exercise training (HIET) can cause adverse effects (renal damage and fibrosis). High concentrations of lactic acid can aggravate renal fibrosis conditions *via* activating TRPV4-TGFβ1-SMAD2/3-CTGF-mediated renal fibrotic pathways in spontaneous hypertension. This finding might provide new ideas for treating hypertensive nephropathy with different intensity exercise in the future.

## Background

The incidence of hypertensive nephropathy continues to rise around the world, and it has become the major leading cause of end-stage renal disease (Udani et al., 2011). From the pathological point of view, the main features of hypertensive nephropathy are inflammation and glomerular and interstitial fibrosis, which ultimately leads to the loss of renal parenchyma (Mennuni et al., 2014). In addition, fibrosis leads to an irreversible exacerbation of compromised function in various organs such as the liver, lungs, heart, and kidney (Rockey et al., 2015).

More and more evidence shows that exercise is an effective non-pharmacological treatment for hypertension and renal fibrosis in cardiovascular diseases (Hagberg et al., 2000; Vina et al., 2012; Huang et al., 2018). However, every coin has two sides; excessive or prolonged high-intensity exercise may damage the health in patients with high blood pressure (Chen et al., 2015a; Ye et al., 2019). During continuous high-intensity exercise, as the rate of glycolysis increases, the final product of anaerobic glycolysis, lactic acid, also increases (Allen et al., 2008).

In the traditional concept, lactic acid is a waste product, which is transported by the blood to the liver and kidneys. However, with the discovery of the Warburg effect in 1920s (Warburg et al., 1927), many studies have found that lactic acid is closely related to tumor growth, immune escape, angiogenesis, and endothelial–mesenchymal transition (San-Millán and Brooks, 2017; Ippolito et al., 2019; Pennington et al., 2019). Furthermore, recent studies suggest that the locally elevated concentration of lactic acid might have a role in promoting cardiac, liver, kidney, pulmonary, and skin fibrosis (Cerbón-Ambriz et al., 1991; van Hall, 2010; Kottmann et al., 2012; Kottmann et al., 2015; Andreucci et al., 2021). Lactic acid is not only the major gluconeogenic precursor but also a vital signaling molecule with endocrine-, paracrine-, and autocrine-like effects and is referred to as a “lactormone” (San-Millan et al., 2022). A recent study has expanded the understanding of the role of lactate acid signaling *via* TGFβ secreted from the adipose tissue of exercising mice (Nikooie and Samaneh, 2016).

Transient receptor potential vanilloid 4 (TRPV4), also known as vanilloid receptor type 4, is a non-selective single-channel cationic current receptor, identified as a transducer of low pH (Ueda et al., 2011; White et al., 2016). Among these profibrotic molecules, TGFβ-1 is one of the most potent inducers of fibrosis pathogenesis, and a growing body of studies showed a close connection between TRPV4 and fibrosis (Rahaman et al., 2014; Wei et al., 2017; Zhan and Li, 2018). As we all know, high-intensity aerobic exercise not only causes the accumulation of lactic acid but also reduces the pH in the cell microenvironment (Robergs et al., 2004). Moreover, many recent studies have found that exercise leads to differential expression of TRPV4 in different tissues (Chen et al., 2015b; Ye et al., 2018).

Although exercise has been proven to be an effective means to lower blood pressure and delay renal fibrosis, some people manifested an increase in blood pressure, rather than reduction after physical exercise (Schlüter et al., 2010). Unfortunately, the impact of different intensity exercise training on renal function is unknown, and the underlying mechanism is also unclear. Our hypothesis is that high-intensity exercise training (HIET) could produce high lactic acid that may aggravate renal inflammatory conditions and activate TRPV4-TGFβ1-Smad2/3-CTGF-mediated renal fibrotic pathways in hypertension, which might cause adverse effects (renal damage and fibrosis).

## Materials and methods

### Animals and experimental protocols

We bought 24 8-week-old male rats from the Vital River Laboratory Animal Technology Co. Ltd. in Beijing, whose weight ranged from 180 to 200 g before the experiment. The 24 male rats are divided into 2 strains: one type is normal blood pressure of Wistar–Kyoto rats (WKY, *n* = 6), and the other is spontaneously hypertensive rats (SHR, *n* = 18). The 24 experimental rats have been fed for 14 weeks, and all of the rats were housed in a temperature-controlled (22–25°C) and humidity-controlled (50%–70%) room with a 12:12 h light–dark cycle. The housing density was 1–5 rats per cage in a specific pathogen-free environment, and rats can get sterilized food and water *ad libitum* in the cages with air exchange. According to the experimental protocol, the selected 24 male rats were randomly assigned to four groups (Udani et al., 2011): the SHR sedentary group (SHR, *n* = 6) (Mennuni et al., 2014), WKY sedentary group (WKY, *n* = 6) (Rockey et al., 2015), the moderate-intensity exercise group (SHR-M, *n* = 6) (the speed is 20 m/min, which is about 50% of the maximum oxygen consumption) (Huang et al., 2018), and high-intensity exercise group (SHR-H, *n* = 6) (the speed is 26 m/min, which is about 65% of the maximum oxygen consumption). After a week of acclimation, according to the experimental plan, the rats are subjected to experimental intervention for 5 days per week (typically 60 min per day) on a rat treadmill (SA101C, Science Monitor, China) with 0°slope. During the 2 days of non-exercise, we used a tail-cuff pressure meter system (BP2010-A; Softron Beijing Biotechnology Co., Ltd.) to continuously measure the heart rate (HR) and the systolic/diastolic blood pressure (SBP/DBP) of 24 male rats and recorded.

All of the experimental procedures were not only approved by the Ethics Committee of Chongqing Medical University but also strictly complied with People’s Republic of China Experimental Animal Regulations (2017 revision) and Chinese animal protection laws and institutional guidelines. Blood samples were collected before the rats were euthanized in accordance with the requirements of national laws.

### Cell culture and treatment

Normal rat kidney interstitial fibroblast cells (NRK-49F) were obtained from Zhejiang Meisen Technology Co. Ltd. (Zhejiang, China). Cells were cultured (37°C, 5% CO2, and 95% relative humidity) in Dulbecco’s modified Eagle’s medium supplemented with 10% fetal bovine serum (Bioagrio) and 1% penicillin−streptomycin (HyClone). The NRK-49F cells were seeded on six-well culture plates to 70% confluence in the complete medium containing 10% fetal bovine serum for 24 h, and the cells were washed three times with a serum-free medium.

After serum starvation for 24 h, the cells were exposed to the treatment for the indicated time periods before harvesting and subjecting them to Western blotting or immunofluorescence staining, respectively. Lactic acid (catalog no. L1750; Sigma-Aldrich) or RN-1734 10 µM (catalog no. 946387-07-1; MedChemExpress) was added to the serum-free medium for indicated time periods and at indicated concentration.

### Western immunoblotting

Cultured NRK-49F cells and renal tissue proteins were extracted using the RIPA Lysis buffer (RIPA, Biyuntian, Hangzhou, China, P0013B) and phenylmethanesulfonyl fluoride (PMSF, Biyuntian, Hangzhou, China, ST505). The homogenates were centrifuged at 12,000 × rpm for 15 min at 4°C, and 5x loading buffer was added to supernatants. Proteins were separated using 10% sodium dodecyl sulfate–polyacrylamide gel electrophoresis gels (PAGE Gel Fast Preparation Kit, Epizyme, China, PG112) and transferred to polyvinylidene difluoride membranes for 2 h at a voltage of 100 V. After blocking with 5% nonfat milk (Cell Signaling Technology, United States) for 2 h at room temperature, the membranes were incubated over 12 h at 4°C. After the membranes were washed three times for 30 min in TBST, the membranes were incubated with the matched secondary antibody for 1 h at room temperature. After using the BeyoECL Moon KIT (Biyuntian, Hangzhou, China, P0018FS), the target bands were quantified using the ChemiDoc Imaging System (Bio-Rad Laboratories, United States) in sequence. The following antibodies were used: anti-TGFβ-1 antibody (1:1000; Abcam, AB179695), anti-Smad2 (phosphoT8) + Smad3 (phosphoT8) (1:1000; Abcam, AB254407), CTGF polyclonal antibody **(**1:500; Proteintech Group, 23936-1-AP), TRPV4 antibody (1:1000; GeneTex, GTX54764), and GAPDH monoclonal antibody (1:5000; Proteintech Group, 60004-1-Ig). Horseradish peroxidase-conjugated goat anti-rabbit and goat anti-mouse antibodies were used as secondary antibodies (1:5000; Proteintech Group).

### Immunohistochemical analyses

Paraffin-embedded kidney sections (4 µm thickness) were stained with Masson’s trichrome staining (Servicebio, China) to assess the degree of fibrosis according to a standard procedure. Afterward, the paraffin-embedded kidney sections were placed in an oven at 60°C for 1 h. Paraffin-embedded specimens were deparaffinized in xylenes for 40 min, rehydrated in different levels of ethanol (100%, 95%, 80%, and 75%) for 20 min, and washed in distilled water and PBS for 10 min. Following antigen retrieval using the microwave thermal repair method for 20 min, the kidney sections were incubated in 3% H_2_O_2_ for 20 min at room temperature. After blocking with blocking solution (10% goat serum) for 30 min at 37°C, the sections delineated by a Dako pen were incubated in specific primary antibodies overnight at 4°C. The kidney sections were washed with PBS five times and incubated with matched secondary antibody for 30 min. After another five washes with PBS, the tissue sections were visualized by dripping with diaminobenzidine, counterstained by hematoxylin, and dehydrated. Immunohistochemistry was performed with antibodies for TRPV4 (1:200; Abcam, AB259361), TGFβ-1 (1:200; Servicebio, GB11179), and anti-rabbit antibody (1:200; Servicebio, G1213-100UL). Also, ImageJ (NIH ImageJ system, Bethesda, MD) was used to analyze images of immunohistochemistry. For each group, three pictures from different samples were taken, quantified, and averaged.

### Immunofluorescence analysis

Cells cultured on coverslips were washed three times with cold 3× PBS and fixed by 4% paraformaldehyde for roughly 15–20 min. After three careful washings with 3× PBS, the cells were treated with 0.5% Triton X-100 for 20 min, blocked with normal goat serum for 30 min at room temperature, and incubated with the anti-TRPV4(1:500; Abcam, AB39260) or anti-TGFβ1 (1:200; Santa, SC-130348) antibody, followed by staining with the tetramethylrhodamine-conjugated secondary antibody for signal amplification in a dark chamber. After the slides were also stained with DAPI to visualize the nuclei, the fluorescent images were acquired using a fluorescence microscope. Pictures of three different experiments were evaluated by three different examiners (SG, TPC, and JCS) for each immunohistochemical staining. For each group, three pictures from different samples were taken, quantified, and averaged.

### Lactate assay

The lactate content in the sera and kidney were measured using the Lactate Determination Kit (Jiancheng, Nanjing, China, A019-2-1). Renal tissue samples were accurately weighed, and normal saline (1 g/9 ml) was added to prepare the renal tissue homogenate. A positive linear relationship was observed between the lactate concentration and the absorbance of the colorimetric product at 530 nm. Briefly, 20 μl of the renal tissue lysate and sera were reacted with 1.2 ml of the luciferase working solution and chromogen solution. The sample buffer was stored in the incubator at 37°C for 10 min, and 2 ml of the termination solution was added to them. The OD of the final mixtures was measured by using a spectrophotometer (Thermo Fisher Scientific, United States) at 530 nm.

### Enzyme-linked immunosorbent assay

The blood and urine of SHR and WKY rats were tested for lipocalin-2/neutrophil gelatinase-associated lipocalin (NGAL) using an enzyme-linked immunosorbent assay (ELISA) kit, according to the corresponding manufacturer’s instructions. NGAL is considered an early and sensitive marker of acute kidney injury. Finally, the optical density value was measured using a spectrophotometer (Thermo Fisher Scientific, United States) at a wavelength of 450 nm; then, we used the results to calculate the lactate concentration. The following ELISA kits were used: rat NGAL ELISA Kit (Boster Biological Technology, Wuhan, China, EK0855) and Rat NGAL ELISA Kit (Jiubang Science & Technology, Quanzhou, China).

### Statistical analysis

All data were compared between WKY, SHR, SHR-M, and SHR-H groups using one-way ANOVA with the GraphPad Prism 8 software. Normally distributed data were analyzed by one-way ANOVA with a *post hoc* Tukey test. For all statistical results, *p* < 0.05 was considered to be statistically significant.

## Result

### Physical and cardiovascular characteristics

There were no significant differences in the initial weight among the four groups. In addition, the final weight in three SHR groups was significantly lower than that in the WKY group. Also, the final weight in the SHR-M and SHR-H group was significantly lower than that in the SHR group. Following the exercise intervention, the systolic blood pressure (SBP), diastolic blood pressure (DBP), and the mean blood pressure (MAP) were lower in the SHR-M group than those in the SHR and SHR-H groups. (all details are presented in Table 1).

**TABLE 1:** Physical characteristics of rats.

Parameter/group	WKY	SHR	SHR-H	SHR-M
Number of animals	6	6	6	6
Initial weight (g)	197 ± 3.0	199 ± 4.4	198 ± 3.5	199 ± 4.1
Final weight (g)	336.±13.4	302 ± 5.1*	276 ± 12.4*△	281 ± 15.0*△
Blood pressure				
Initial SBP (mm Hg)	96 ± 5.0	147 ± 3.3*	144 ± 1.8*	143 ± 1.9*
Final SBP (mm Hg)	108 ± 5.9	157 ± 8.3*	158 ± 7.3*	140 ± 4.3*△#
Initial MAP (mm Hg)	75 ± 4.0	129 ± 2.1*	130 ± 1.6*	127 ± 1.5*
Final MAP (mm Hg)	91 ± 3.7	140 ± 6.3*	144 ± 6.7*	125 ± 4.3*△#
Initial DBP (mm Hg)	65 ± 5.6	121 ± 2.7*	122 ± 2.1*	119 ± 2.0*
Final DBP (mm Hg)	82 ± 4.8	132 ± 6.0*	136 ± 6.7*	117 ± 5.9*△#
Initial HR (beats per min)	362 ± 28.2	435 ± 17.0*	445 ± 9.6*	440 ± 13.2*
Final HR (beats per min)	373 ± 36.5	426 ± 20.8*	430 ± 20.5*	412 ± 34.1

### Effects of different intensity exercise training on blood pressure

**FIGURE 1:**
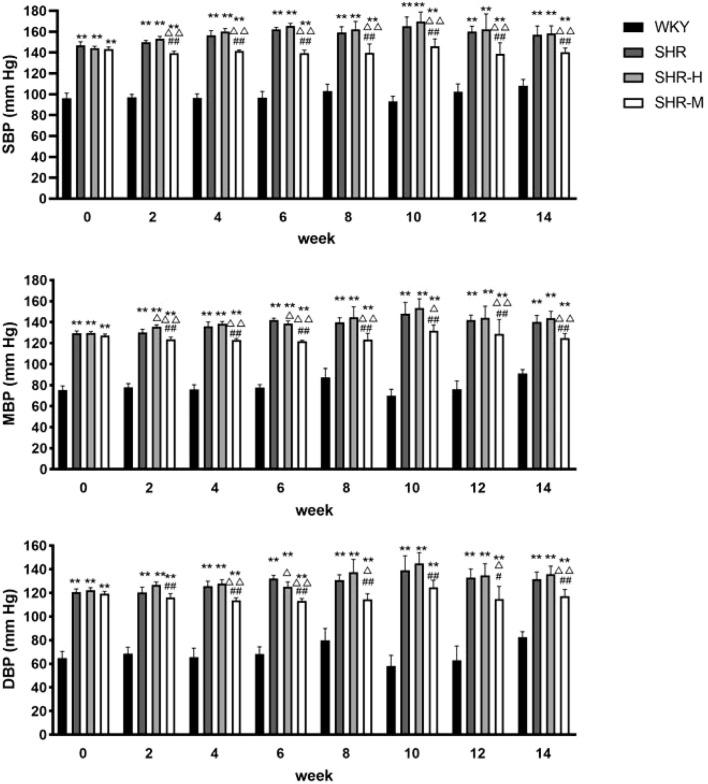
Blood pressure statistics for 14 consecutive weeks. Evaluation of MBP, SBP, and DBP at baseline (week 0) and in weeks 2, 4, 6, 8, 10, 12, and 14 of the protocol. ^*^ *p* < 0.05 and ^**^ *p* < 0.01 versus the WKY group. ^△^ *p* < 0.05 and ^△△^ *p* < 0.01 versus the SHR group. ^#^ *p* < 0.05 and ^##^ *p* < 0.01 versus the SHR-H group.

To investigate whether exercise training alleviates hypertension in SHR or not, we used a caudal cuff method to measure blood pressure every 2 weeks. At the beginning of the experiment, the systolic blood pressure (SBP), the mean blood pressure (MAP), and the diastolic blood pressure (DBP) of SHR were much higher than WKY. Starting from the second week, the SHR-M group had significant statistical differences compared with the other two groups (SHR and SHR-H), especially in SBP. Moreover, the SHR-M group is statistically different from the SHR-H group after the exercise intervention started. By contrast, HIET did not lead to any changes in SBP, MBP, and DBP in SHR (Figure 1).

### Effects of different intensity exercise training on renal tissue architecture and fibrosis

**FIGURE 2:**
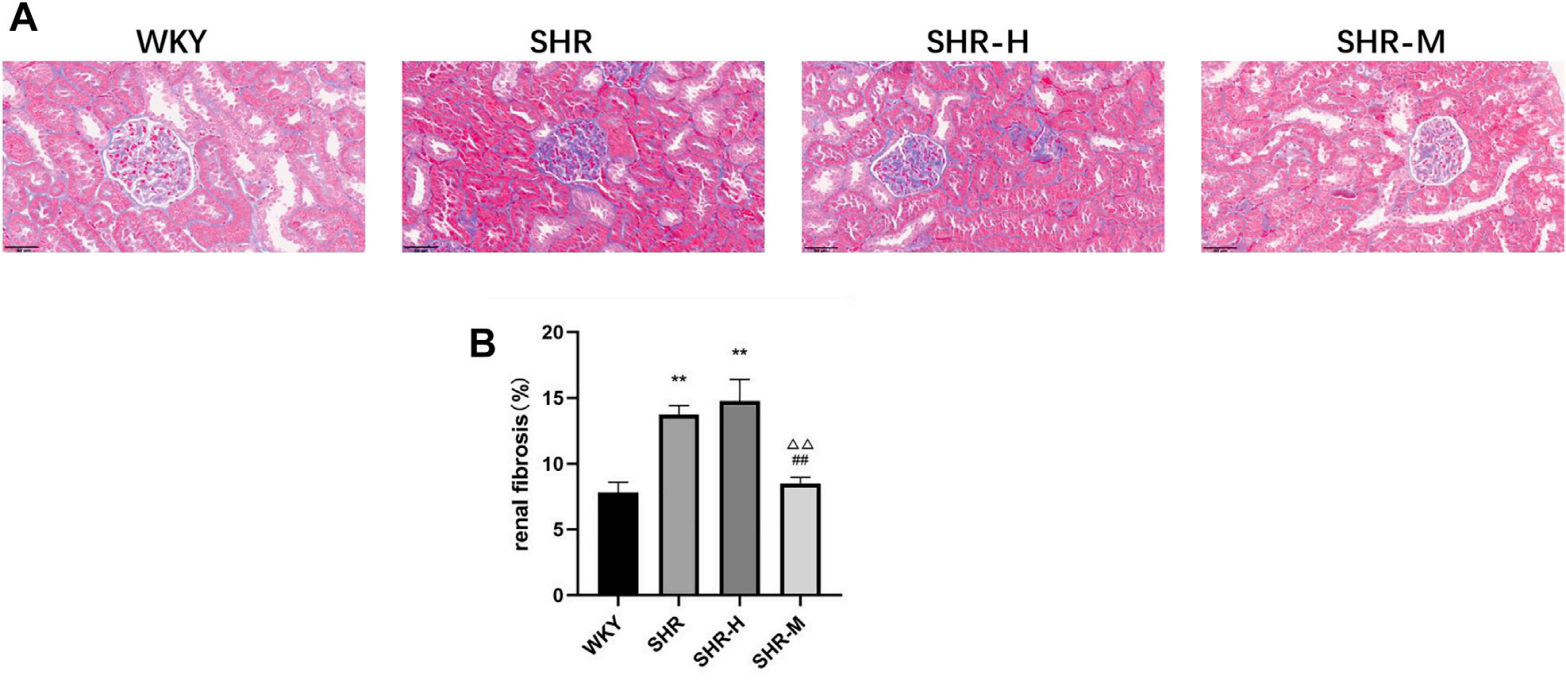
Effects of different intensity exercise training on renal tissue architecture and fibrosis. **(A)** Paraffin-embedded kidney sections (4 µm thickness) were stained with Masson’s trichrome stain (scale bar = 20 µm). **(B)** Fibrosis area was calculated and shown by a column graph. All images were taken at ×400 magnification. Values are mean ± S. D (*n* = 3 in each group). ^*^ *p* < 0.05 and ^**^ *p* < 0.01 versus the WKY group. ^△^ *p* < 0.05 and ^△△^ *p* < 0.01 versus the SHR group. ^#^ *p* < 0.05 and ^##^ *p* < 0.01 versus the SHR-H group.

To evaluate whether there were changes in renal fibrosis after exercise training of different intensities, the histology of renal cortical slices was analyzed by Masson’s trichrome staining. As shown in Figures, we found that the renal cortices in the SHR group and the SHR-H group held a much larger area of fibrosis than the WKY group and the SHR-M group, whereas no significant difference was observed in the SHR group and the SHR-H group (Figures 2A,B).

### Effects of different intensity exercise training on renal function

To further observe the severity of kidney damage, the levels of the renal injury marker NGAL, BUN, creatinine, and urine protein in the plasma and urine were measured. The results showed that urine NGAL, urine protein, plasma NGAL, plasma BUN, and plasma creatinine did not differ between the SHR-H group and the SHR group. Significantly lower levels of urine protein, urine NGAL, plasma NGAL, plasma BUN, and plasma creatinine were observed in the SHR-M group than in the SHR group and the SHR-H group at the end of the study. Interestingly, the results revealed that the levels of plasma creatinine, plasma BUN, and urine protein in the SHR-M group were significantly lower than those in the SHR group.

### Effects of different intensity exercise training on lactic acid

To explore whether there is a link between lactic acid and fibrosis, we measured the total lactate level using a commercial kit. The results revealed that renal lactic acid was significantly higher in the SHR group and the SHR-H group than that in the WKY group. Moreover, renal lactic acid was significantly higher in the SHR-H and SHR groups than that in the SHR-M group. The results revealed that blood lactic acid in the SHR-M group was significantly lower than that in the SHR-H group and the SHR group.

### Effects of different intensity exercise training on related fibrotic molecules

**FIGURE 3:**
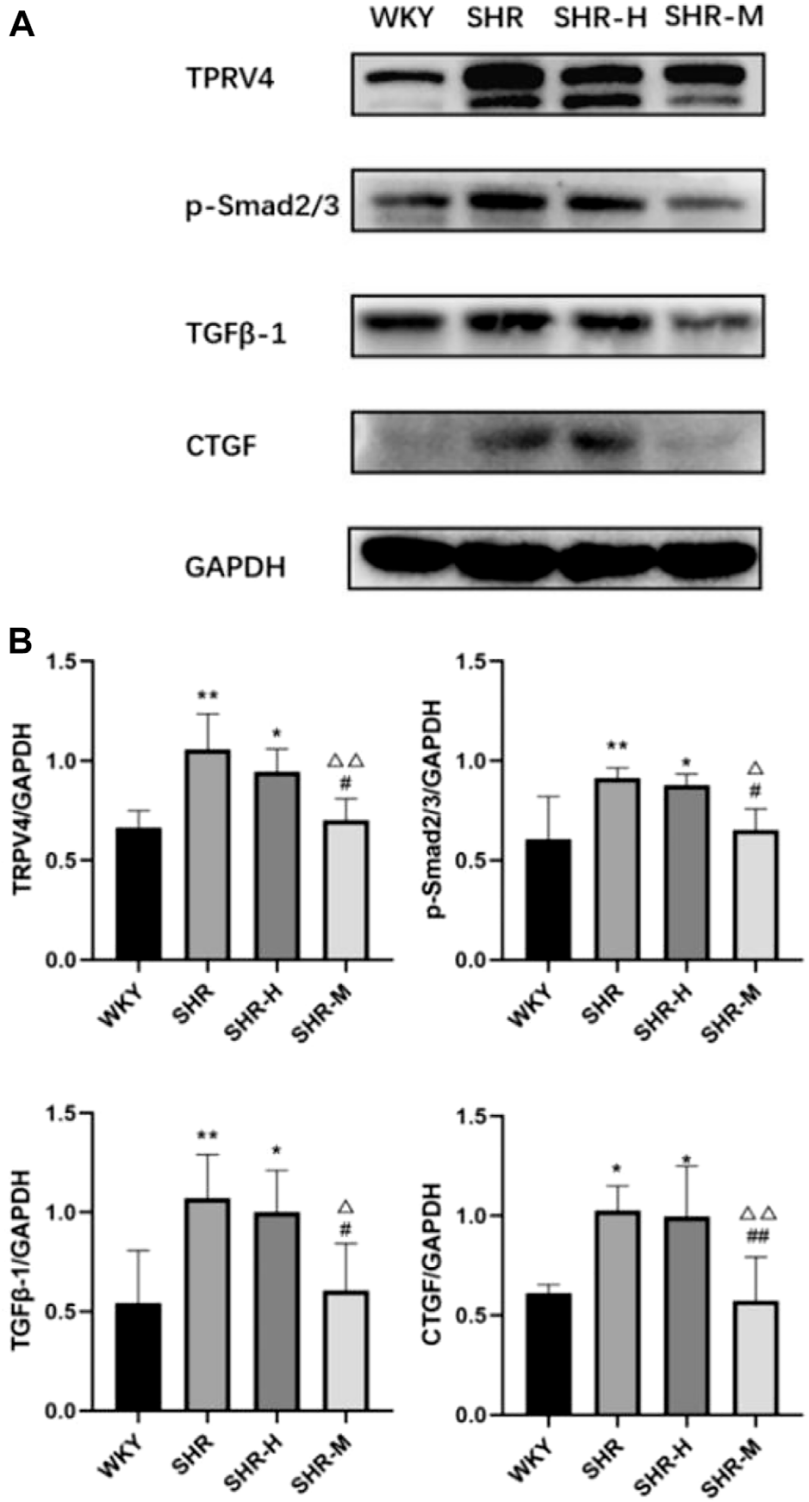
Effects of different intensity exercise training on related fibrotic molecules. **(A)** Western blotting result and responding quantification of protein products of transient receptor potential vanilloid 4 (TRPV4), transforming growth factor-beta (TGFβ-1), anti-Smad2 (phosphoT8) + Smad3 (phosphoT8) (p-Smad2/3), and connective tissue growth factor (CTGF) extracted from the renal tissue. **(B)** Bars represent the corresponding protein quantification of TRPV4, TGFβ-1, p-Smad2/3, and CTGF on the basis of GAPDH. Values are mean ± S.D. (*n* = 5 in each group). ^*^ *p* < 0.05 and ^**^ *p* < 0.01 versus the WKY group. ^△^ *p* < 0.05 and ^△△^ *p* < 0.01 versus the SHR group. ^#^ *p* < 0.05 and ^##^ *p* < 0.01 versus the SHR-H group.

**FIGURE 4:**
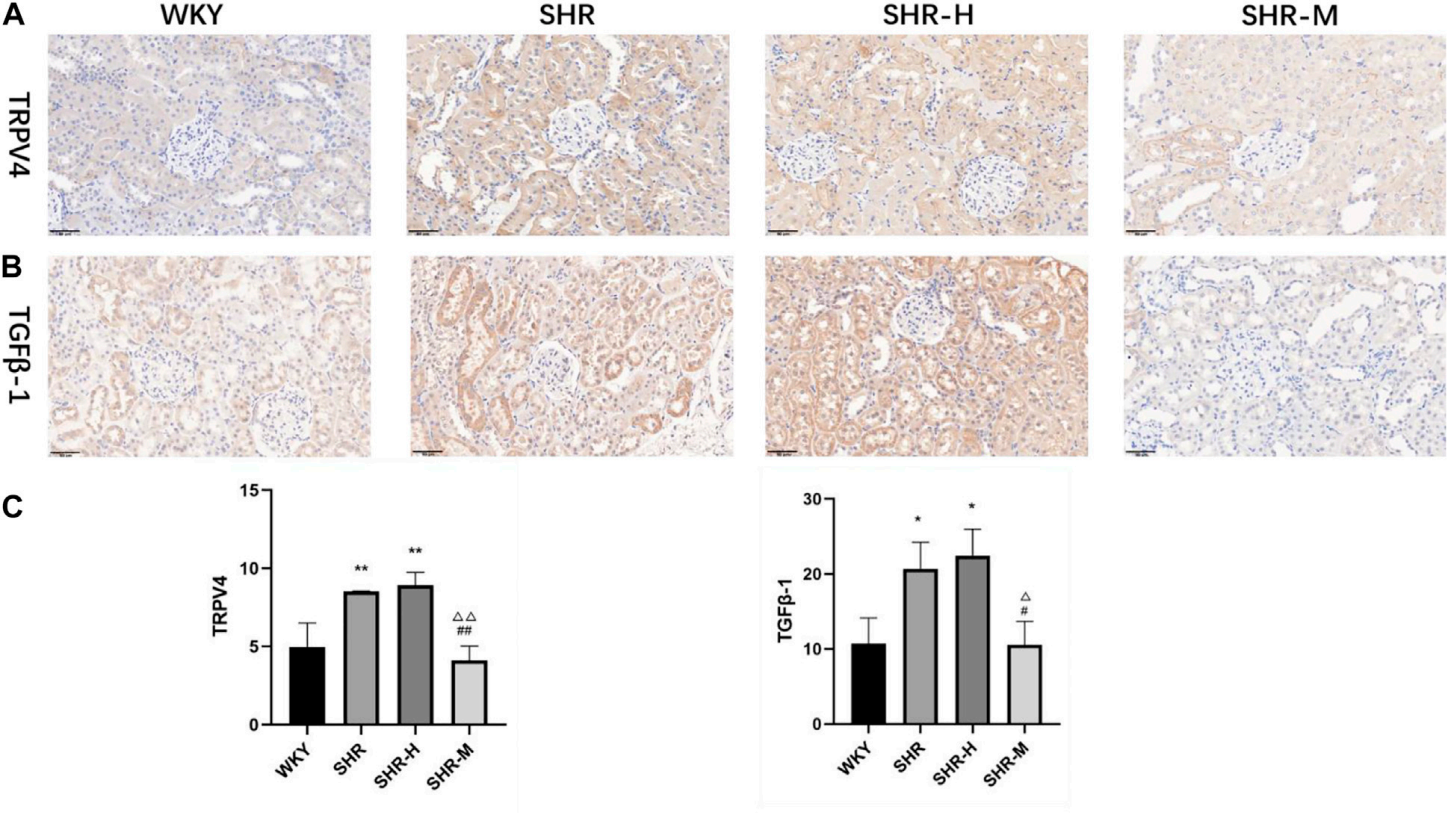
Effects of different intensity exercise training on related fibrotic molecules. **(A,B)** Immunohistochemical staining result and responding quantification of protein products of transient receptor potential vanilloid 4 (TRPV4) and transforming growth factor-beta (TGFβ-1). **(C)** Bars represent the corresponding protein quantification of TRPV4 and TGFβ-1. Values are mean ± S. D (*n* = 3 in each group). ^*^ *p* < 0.05 and ^**^ *p* < 0.01 versus the WKY group. ^△^ *p* < 0.05 and ^△△^ *p* < 0.01 versus the SHR group. ^#^ *p* < 0.05 and ^##^ *p* < 0.01 versus the SHR-H group.

To further investigate the renal fibrotic signaling pathway in hypertensive models with different exercise training, the protein levels of TRPV4, the TGFβ-1, p-Smad2/3, and CTGF in the renal cortex from the WKY, SHR, SHR-M, and SHR-H groups were measured. When compared with the SHR and SHR-H groups, the key fibrotic protein levels of TRPV4, TGFβ-1, p-Smad2/3, and CTGF were significantly decreased in the SHR-M group (Figure 3). We also found that the expression levels of lactic acid-related TRPV4 and TGFβ-1 obtained by immunohistochemical analysis (Figure 4C) were similar to those obtained by Western blot analysis (Figure 3B).

### Effects of lactic acid on related fibrotic molecules *in vitro*

**FIGURE 5:**
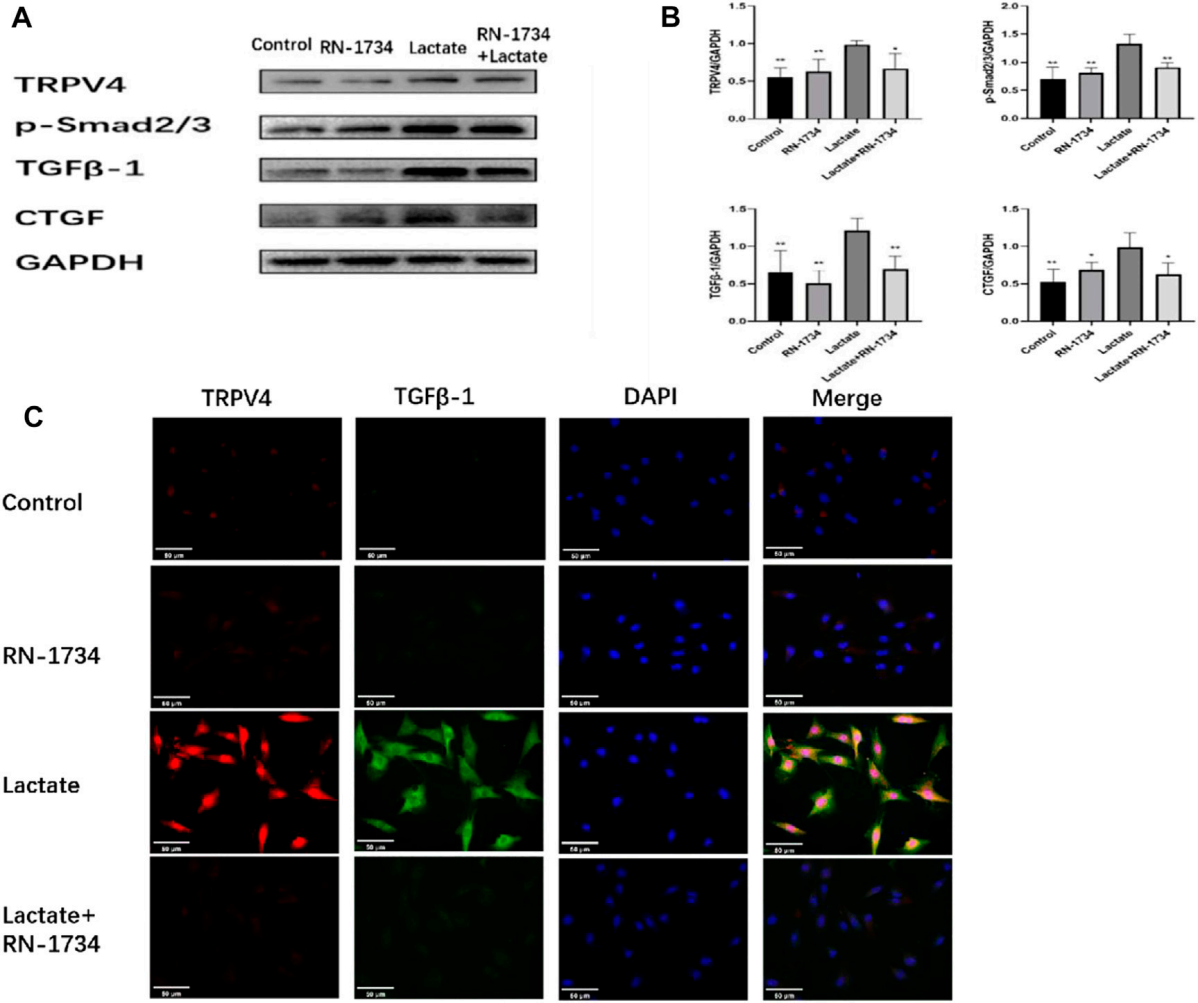
Effects of lactic acid on related fibrotic molecules *in vitro*. **(A)**Western blotting result and responding quantification of protein products of transient receptor potential vanilloid 4 (TRPV4), transforming growth factor-beta (TGFβ-1), anti-Smad2 (phosphoT8) + Smad3 (phosphoT8) (p-Smad2/3), and connective tissue growth factor (CTGF) extracted from the rat kidney interstitial fibroblast (NRK-49F) cells. **(B)** Bars represent the corresponding protein quantification of TRPV4, TGFβ-1, p-Smad2/3, and CTGF on the basis of GAPDH. Values are mean ± S.D. (*n* = 5 in each group). ^$^ *p* < 0.05 and ^$$^ *p* < 0.01 versus the lactate group. **(C)** Immunofluorescence staining of responding protein products of transient receptor potential vanilloid 4 (TRPV4) and transforming growth factor-beta (TGFβ-1).

To further investigate the renal fibrotic signaling pathway *in vitro*, the protein levels of TRPV4, TGFβ-1, p-Smad2/3, and CTGF were measured in the NRK-49F cells. When compared with the control, RN-1734, and Lactate + RN-1734 group, the key fibrotic protein levels of TRPV4, TGFβ-1, p-Smad2/3, and CTGF were significantly increased in the lactate group (Figures 5A,B). We also found that the expression levels of lactic acid-related TRPV4 and TGFβ-1 obtained by immunofluorescence staining results (Figure 5C) were similar to those obtained by Western blot results (Figure 5A).

## Discussion

**FIGURE 6:**
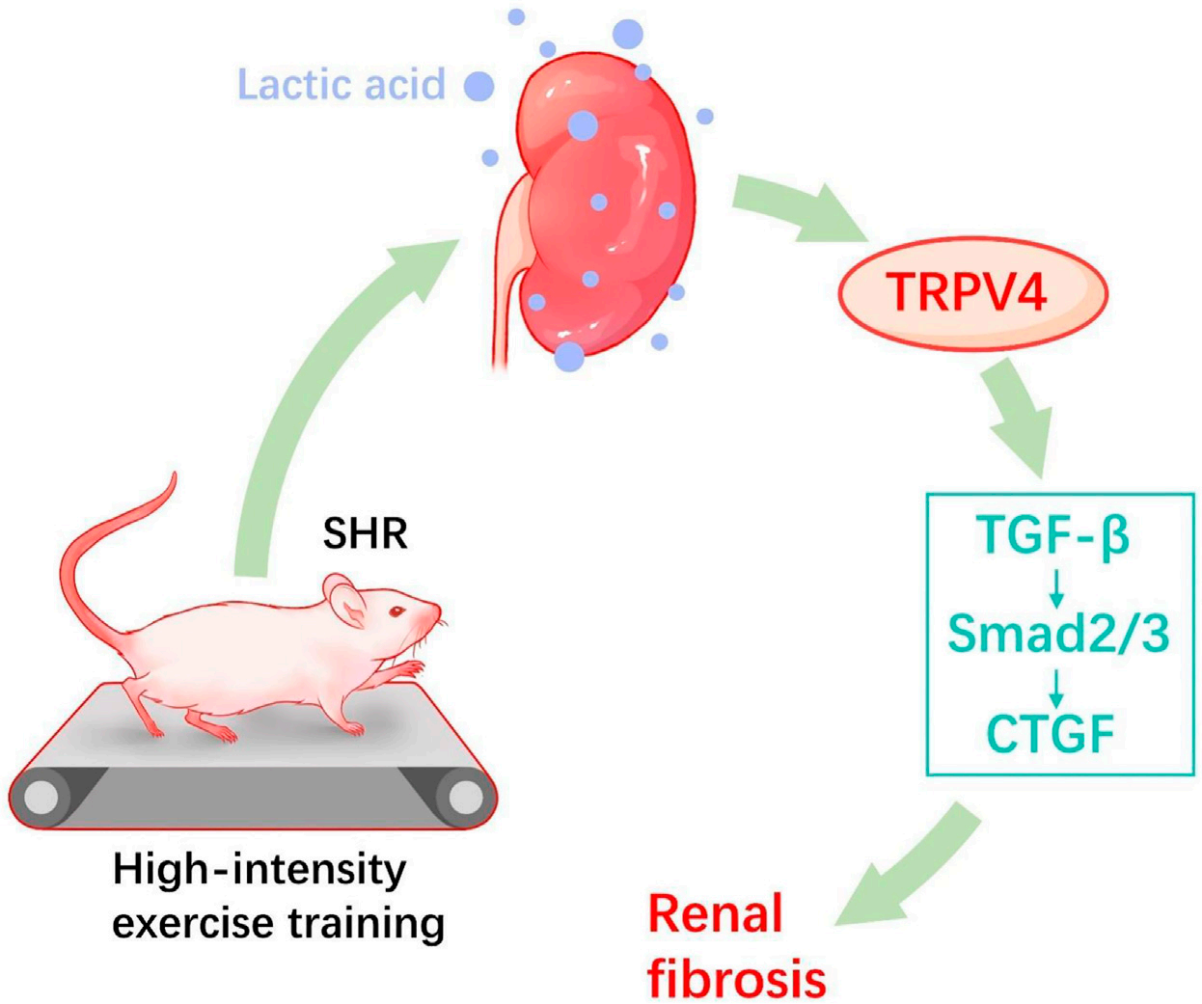
Proposed hypothesized diagram. High-intensity exercise training appears to aggravate the hypertension-induced renal fibrotic pathway (TRPV4, TGF-β, p-Smad2/3, and CTGF) and lactate accumulation in hypertensive rat models.

In summary, the study demonstrates that HIET exacerbated hypertension-associated renal dysfunction and fibrosis, whereas moderate-intensity exercise reduced the blood pressure and reversed renal function. Our data also demonstrate that moderate-intensity exercise training reduced renal and blood lactate in SHR. However, instead of a rather positive effect, opposite results were observed in the SHR-H group. Here, TRPV4 and TGFβ-smad2/3 signal pathway activation by lactate may play a vital task in hypertension-enhanced renal fibrosis (Figure 6).

At present, the development of chronic kidney disease (CKD) has been regarded as a cause of the worsening prognosis due to cardiovascular morbidity and mortality (Segura et al., 2004). Renal fibrosis is the final common pathological outcome of the progression of many renal diseases, which will lead to irreversible injury to renal function, ultimately leading to renal failure (Humphreys, 2018). To date, there is a lack of effective targeted therapies for pathological fibrosis. At present, RAAS inhibitors (ACEIs and ARBs) are recommended as the first-line medication in the clinical practice for hypertensive patients with renal disease, which can cause all kinds of adverse reactions such as excessive blood pressure reduction or elevated blood potassium levels (in the elderly patients), resulting in withdrawal of RAAS inhibitors in the case of acute worsening of renal failure (Parving et al., 2012).

Currently, in preclinical studies, pirfenidone and fresolimumab are considered as a potential therapeutic way for renal fibrosis, which is the direct blockade of the TGFβ-Smad2/3 classical signaling pathway with antagonists or inhibitors (Fan et al., 1999; Xavier et al., 2015). However, if this classical TGFβ-Smad2/3 signaling pathway is blocked, the disruption of anti-inflammatory and antitumor properties may be a major barrier for potential treatments (Meng et al., 2015). Thus, reasonable exercise and healthy diet as an alternative non-pharmaceutical means to protect the kidney have been commonly advocated. In the past, studies have suggested that moderate-intensity exercise training decrease blood pressure and prevent renal fibrosis in hypertensive rats (Huang et al., 2018). Similarly, Huang et al. (2018) found that the SBP, renal fibrosis area, and the fibrotic-related protein levels of TGFβ-1, p-Smad2/3, and CTGF were significantly decreased in the moderate-intensity exercise training group compared to the SHR group, but our current findings showed adverse effect in the SHR-H group. HIET modalities have different effects on endothelial dysfunction and ultrastructural remodeling, mesenteric arterial remodeling, and cardiac function in patients with hypertension (Chen et al., 2015a; Ye et al., 2019). However, little public information has been obtained on the effects of SHR with HIET on renal function.

**FIGURE 7:**
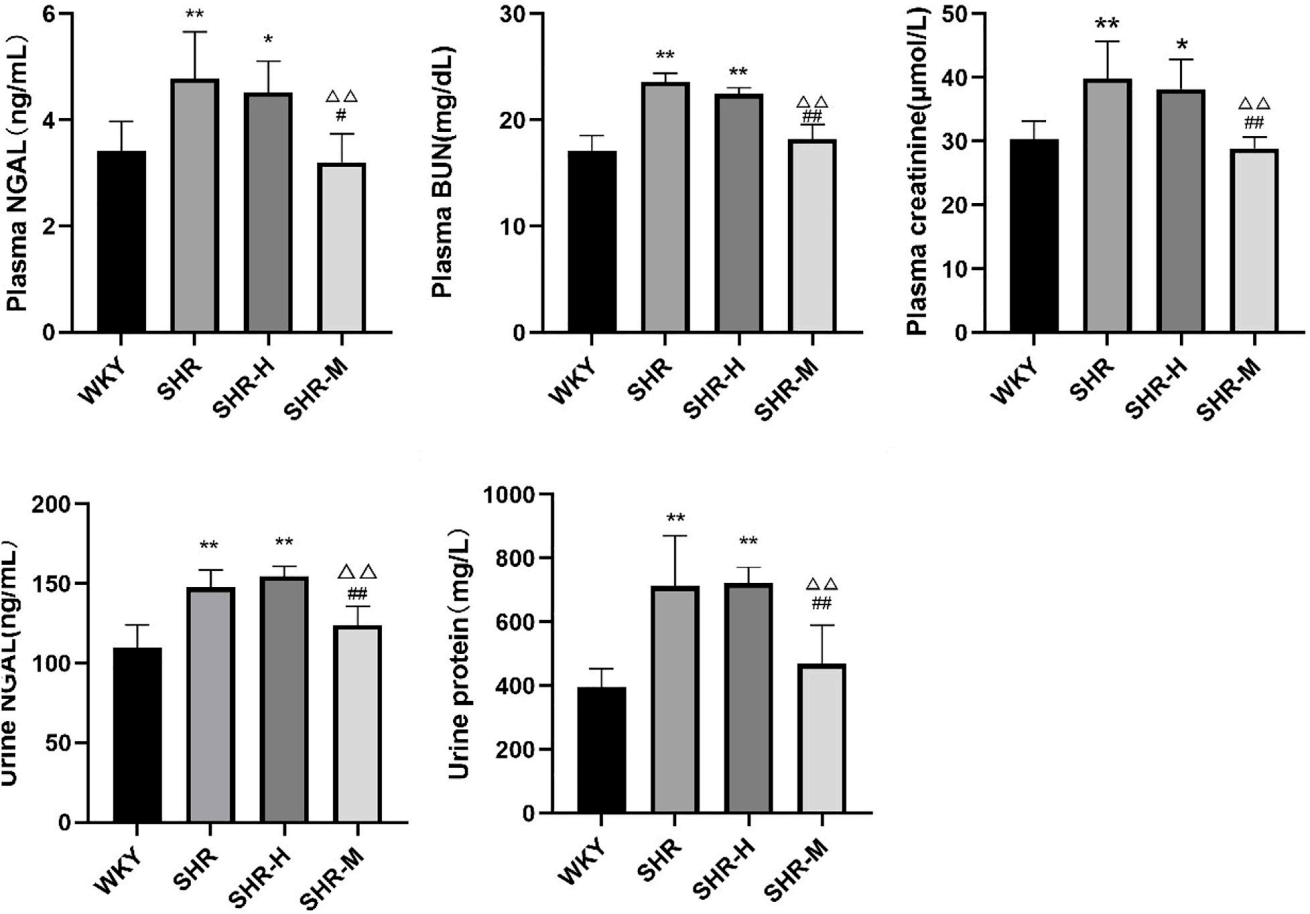
Effects of different intensity exercise training on renal function. NGAL, neutrophil gelatinase-associated lipocalin; BUN, blood urea nitrogen. The histograms reflect the content of the kidney injury index and indicate the mean values ±SD (*n* = 6 in each group). ^*^ *p* < 0.05 and ^**^ *p* < 0.01 versus the WKY group. ^△^ *p* < 0.05 and ^△△^ *p* < 0.01 versus the SHR group. ^#^ *p* < 0.05 and ^##^ *p* < 0.01 versus the SHR-H group.

**FIGURE 8:**
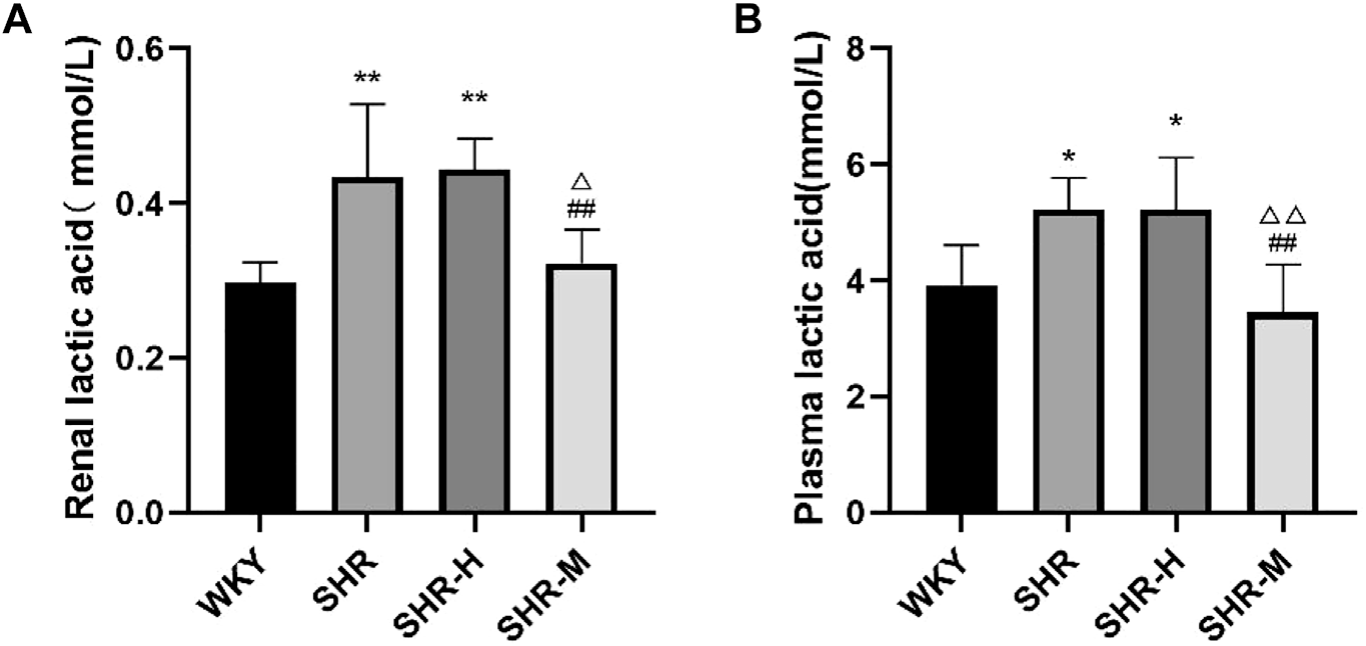
Effects of different intensity exercise training on lactic acid. **(A,B)** Histograms reflect the concentration of lactic acid and indicate mean values ±SD (*n* = 6 in each group). ^*^ *p* < 0.05 and ^**^ *p* < 0.01 versus the WKY group. ^△^ *p* < 0.05 and ^△△^ *p* < 0.01 versus the SHR group. ^#^ *p* < 0.05 and ^##^ *p* < 0.01 versus the SHR-H group.

Based on our experiments, by observing the levels of biomarkers such as blood creatinine, urea nitrogen, NGAL, and urine protein, we found that renal function appeared to deteriorate rapidly after HIET (Figure 7). Not only did we find that evidence for the expression level of TRPV4 and TGFβ-1 was greater in the SHR-H group and SHR group but also the same phenomenon occurred in renal and blood lactic acid (Figure 8). This could be caused by the increase of gluconeogenesis after the renal function improved, which led to a decrease in the accumulation of lactic acid in the kidneys. Under fasting and stress conditions, renal gluconeogenesis accounts for 40%, and it has been discovered that renal glucose and lactate metabolism are closely related to mortality associated with acute kidney injury (Legouis et al., 2020). It was observed that the expression concentration of lactic acid was correlated with the expression levels of TGFβ-1 and TRPV4. Kottmann et al. have identified that lactic acid as a glycolytic metabolite was an important media of myofibroblast differentiation by a pH-dependent activation of TGF-β (15). In addition, they also found that gossypol as the pharmacologic inhibition of the lactate dehydrogenase activity can suppress TGF-β-induced myofibroblast differentiation by the suppression of extracellular accumulation of lactic acid and the suppression of TGF-β bioactivity in 2015 (Kottmann et al., 2015).

Although maximal lactate steady state was recently proposed in spontaneously hypertensive rats as an important step in establishing suitable intensities for prescribing exercise for hypertensive patients (Hidayat et al., 2017), we found a significant difference in blood lactate until 24 h after the last exercise, and blood lactate may be an indicator for renal function in hypertensive patients. In addition, renal lactate elevation was found to be strongly associated with the increased expression of TRPV4 and TGFβ-1. Through prolonged exercise training after 14 weeks, we can accurately reflect the results of BP, SBP, DBP, HR, and renal function after long-term exercise, and we found that HIET can cause adverse effects (renal damage and fibrosis).

In summary, lactate is closely correlated with TRPV4 and TGFβ-1 as key signaling molecules in fibrosis formation. It is clear that controlling TRPV4 and lactate is an effective way for hypertensive phenotypes to inhibit renal fibrosis. Clinically, blood lactate after exercise may be a useful tool for prescribing exercise for hypertensive phenotypes. Therefore, these exercises’ instruction may not be generalized for diabetic patients, patients with a tumor, and those with chronic fibrotic disease.

With the aging population, the incidence of hypertension and cardiovascular-related events continues to increase worldwide, and morbidity and cancer‐specific mortality continue to increase in many countries. Due to the continuous development of novel drugs, the number of hypertensive patients with tumor appears to increase. However, such an effort is currently limited due to the lack of relevant experimental and clinical data about optimal blood pressure control strategy for tumor patients with hypertension. Epidemiological studies in recent years have shown that hypertension was significantly associated with an increased risk of kidney cancer, colon cancer, and postmenopausal breast cancer (Han et al., 2017; Hidayat et al., 2017; Sud et al., 2018). Similarly, vascular endothelial growth factor signaling pathway inhibitors as common antineoplastic drugs are key contributors to raise blood pressure (Agarwal et al., 2018). Lactate is the end-product of glycolysis and is overproduced in many cancer cells under the Warburg effect (Warburg, 1956). At the same time, TRPV4 differential expression has also been closely related to the onset and progression of many tumors, and TRPV4 may be a target for cancer diagnosis and treatment (Yu et al., 2019). According to these characteristics, a growing body of evidences also showed that TRPV4 acted as a vital regulator in the progression of tissue-specific fibrotic diseases including lung, liver, kidney, heart, and skin fibrosis, suggesting that TRPV4 may be a potential therapeutic target (Zhan and Li, 2018). Thus, the inhibition of lactic acid and TRPV4 is applicable to hypertensive patients with cancer as a means to uncover disease mechanisms and discover potential therapeutic targets.

In addition, previous studies have shown that aerobic exercise is an effective way to regulate renal fibrosis and reduce blood pressure. Since different exercise intensities have different results, different exercise methods also have different results. In recent years, high-intensity interval training (HIIT) and resistance exercise have proposed new forms of fitness. During natural aging, HIIT does not protect against heart damage compared to moderate-intensity exercise training (Pei et al., 2021). The study found that HIIT is superior to continuous training in the treatment of right ventricular hypertrophy and dysfunction caused by pulmonary hypertension (Brown et al., 2017). HIIT and resistance training have opposite effects on markers of heart failure and cardiac remodeling in hypertensive rats (Holloway et al., 2015). Aerobic exercise training and resistance training alleviate cardiac fibrosis and cardiac insufficiency after myocardial infarction *via* the TGFβ-Smad pathway, respectively (Ma et al., 2021). Combined aerobic and resistance exercise training improves menopause-related hypertension (Shimojo et al., 2018). Different intensities and different exercise modes are difficult to control the therapeutic effect of the disease. Lactic acid, as an exercise metabolite, has been listed as a measure of resistance exercise aerobic metabolism (Domínguez et al., 2018). Also, the study also found that HIIT-induced lactate accumulation modulates intramuscular triglyceride metabolism *via* a transforming growth factor-β1-mediated pathway (Nikooie and Samaneh, 2016).

At this stage, compared with one type of exercise therapy, we are gradually realizing the significance of combining multiple trainings in the treatment of diseases. Lactic acid may become a marker for defining various exercise training methods. This finding might provide new ideas for treating hypertensive nephropathy and other diseases with different intensity exercise in the future. Our research group plans to explore the mechanism of combined exercise training in the treatment of hypertension.

## Data Availability

The original contributions presented in the study are included in the article/Supplementary Material. Further inquiries can be directed to the corresponding author.
